# Comment on Levitt et al. Approach to Decompensated Right Heart Failure in the Acute Setting. *J. Clin. Med.* 2024, *13*, 869

**DOI:** 10.3390/jcm13133848

**Published:** 2024-06-29

**Authors:** Carolina Brea, Ellen Freeh, Michael I. Prats

**Affiliations:** Department of Emergency Medicine, The Ohio State University, Columbus, OH 43210, USA; ellen.freeh@osumc.edu (E.F.);

We read with great interest the article titled “Approach to Decompensated Right Heart Failure in the Acute Setting” [1]. We admire the authors’ thorough review of the pathophysiology, morbidity and mortality, diagnostic modalities, and treatment options relating to acute right heart failure (ARHF). This is certainly a topic that merits close attention by many medical providers as it can pose significant risk to our patients. Of particular interest was the use of point-of-care ultrasound (POCUS) in the diagnosis of ARHF at bedside. In this article, a plethoric inferior vena cava (IVC) was described as a diameter <10 mm without inspiratory collapse. We feel that this definition is not in accordance with the standard definition, and in general, an IVC diameter > 20 mm with <50% collapse more accurately defines a plethoric IVC [2,3,4,5,6,7,8,9,10,11].

The term plethoric originates from the Greek *plethein*, meaning “to be full”, and was used by physicians as early as Hippocrates to describe excesses of bodily fluids [12]. In 1988, one of the earliest published studies to use the term describes a “plethora of the inferior vena cava”, defined as an IVC with <50% decrease in diameter after deep inspiration [10]. It was associated with elevated central venous pressure and found to be a sensitive marker of cardiac tamponade. Since there have been countless studies that examine the value of IVC measurements with POCUS in the determination of right atrial (RA) or central venous pressures. Perhaps one of the most referenced standards is the American Society of Echocardiography guideline for the assessment of the right heart, which defines an IVC diameter > 2.1 cm that collapses < 50% with a sniff as suggesting high RA pressure between 10–20 mm Hg [8]. Given that patients who are on positive pressure ventilation will have distention of their IVC from positive pressure during inspiration, it should be recognized that in this case, plethoric could be used to describe an IVC without significant distensibility [9]. Given the potential for ambiguity in the use of plethoric as a descriptor for the IVC, it might be preferrable to specifically describe the diameter and respiratory variation to accurately convey the sonographic findings.

Overall, the interpretation of the IVC is intricate and necessitates a wholistic understanding of the patient’s condition [11]. If the term plethoric is used, there should be consistency in the definition and implications. As the authors stated, ARHF is complex, with pathophysiology varying by the etiology of the disease process, and several data points are likely needed for its accurate diagnosis. We agree with the authors that POCUS is a valuable tool for the rapid diagnosis of ARHF and can help guide management. IVC diameter and respiratory variation can help as one component of the evaluation in the determination of right atrial or central venous pressure. In accordance with the preponderance of the literature, a plethoric IVC in this case should be one with a diameter > 20 mm with <50% collapsibility.
